# Conophylline inhibits non-alcoholic steatohepatitis in mice

**DOI:** 10.1371/journal.pone.0178436

**Published:** 2017-06-08

**Authors:** Yukiomi Nakade, Kazumasa Sakamoto, Taeko Yamauchi, Tadahisa Inoue, Yuji Kobayashi, Takaya Yamamoto, Norimitsu Ishii, Tomohiko Ohashi, Yoshio Sumida, Kiyoaki Ito, Haruhisa Nakao, Yoshitaka Fukuzawa, Kazuo Umezawa, Masashi Yoneda

**Affiliations:** 1 Division of Gastroenterology and Hepatology, Department of Internal Medicine, Aichi Medical University, Nagakute, Aichi, Japan; 2 Department of Molecular Target Medicine Screening, Aichi Medical University, Nagakute, Aichi, Japan; INRA, FRANCE

## Abstract

Conophylline (CnP), a vinca alkaloid extracted from the leaves of the tropical plant *Ervatamia microphylla*, attenuates hepatic fibrosis in mice. However, little is known about whether CnP inhibits steatosis, inflammation, and fibrosis in non-alcoholic steatohepatitis (NASH) in mice. A methionine-choline-deficient (MCD) diet was administered to male *db/db* mice as a NASH model, and CnP (1 μg/kg/d) was co-administered. Eight weeks after the commencement of the MCD diet, hepatic steatosis, inflammation, and fibrosis, and hepatic fat metabolism-, inflammation-, and fibrosis-related markers were examined. Feeding on an MCD for 8 weeks induced hepatic steatosis, inflammation, and fibrosis. CnP significantly attenuated the MCD-induced increases in hepatic steatosis, as well as hepatic inflammation and fibrosis. The MCD diet increased hepatic transforming growth factor-β (TGF-β) mRNA levels, which are correlated with hepatic steatosis, inflammation, and fibrosis. The diet also attenuated acyl-coenzyme A oxidase 1 (ACOX1) and carnitine palmitoyltransferase 1 (CPT1) mRNA levels, which are involved in β-oxidation. The putative mechanism of the CnP effect involves reduced hepatic TGF-β mRNA levels, and increased mRNA levels of hepatic peroxisome proliferator-activated receptor (PPAR) α and its target genes ACOX1 and CPT1. The results of this study indicate that CnP inhibits steatohepatitis, possibly through the inhibition of hepatic TGF-β mRNA levels, and induces an increase in PPARα mRNA levels, resulting in the attenuation of hepatic steatosis, inflammation, and fibrosis in mice. CnP might accordingly be a suitable therapeutic option for NASH.

## Introduction

Non-alcoholic fatty liver disease (NAFLD), which encompasses a wide range of disorders, has become a major health issue and the most common liver disease throughout the world [1]. NAFLD is considered to be the hepatic manifestation of metabolic syndrome, which is reported to be a cluster of cardiovascular risk factors [2–4]. Non-alcoholic steatohepatitis (NASH), which is characterized by steatosis, necroinflammation, cytopathic changes, and liver fibrosis, finally develops to liver cirrhosis [5]. Although the pathogenesis of NASH remains to be elucidated, insulin resistance and obesity are considered to play important roles in NASH progression. Since there is currently no effective therapy for NASH, it desirable to identify a new avenue for the regulation of steatohepatitis [6, 7].

Conophylline (CnP), a vinca alkaloid extracted from the leaves of the tropical plant *Ervatamia microphylla* [8], has been shown to mimic the effect of activin A, since both induce β-cell differentiation in precursor cells [9]. However, activin A is an autocrine activator of pancreatic stellate cells and increases the expression of α-smooth muscle actin (α-SMA) and collagen [10], whereas CnP has the opposite effect on the activation of pancreatic stellate cells, by inhibiting their growth, and reduces the expression of α-SMA and collagen *in vitro* [11]. Previous study has shown that CnP also improves islet fibrosis in diabetic rats, which are animal models of type 2 diabetes [11]. With regards to the hepatobilliary system, CnP suppresses hepatic stellate cells and attenuates experimental hepatic fibrosis in rats [12]; however, it remains to be elucidated whether CnP attenuates steatohepatitis in mice.

In experimental animal studies, high-fat and/or high-cholesterol diets have been used as obese NAFLD models [13, 14], whereas a methionine-choline-deficient (MCD) diet has been reported to induce steatohepatitis, which is morphologically similar to NASH [15]. An MCD diet has been shown to induce adipose tissue lipolysis, resulting in an increase in serum-free fatty acid (FFA) levels and an increase in hepatic triglycerides (TG), and also gives rise to macrovesicular steatosis and enhances inflammation in the liver within a few weeks [16, 17]. Although an MCD diet decreases body weight and insulin resistance in mice, it induces steatohepatitis and liver fibrosis within 8 weeks, providing a useful rodent model for pathological NASH [15, 18]. *db/db* mice spontaneously develop type 2 diabetes and fatty liver with obesity due to a partial functional defect in the long form of the leptin receptor [19]. Furthermore, these mice are hyperphagic when a high-fat or normal diet is administered, and hepatic steatosis in *db/db* mice is significantly increased compared with that in control mice, even when a normal diet is administered [18]. When fed an MCD diet, *db/db* mice have been reported to develop hepatic steatosis, inflammation, and fibrosis [15].

In the present study, we examined whether CnP decreases hepatic steatosis, inflammation, and fibrosis in *db/db* mice fed an MCD diet. We demonstrate that CnP improves steatohepatitis in mice via the downregulation of transforming growth factor-β (TGF-β) and upregulation of peroxisome proliferator-activated receptor α (PPARα) and its downstream targets involved in fatty acid oxidation.

## Materials and methods

### Substances and treatments

CnP was isolated and purified from the leaves as described previously [8]. The leaves were collected from *Tabernaemontana divaricata*, cultivated in Miyako-jima island located at 24 degrees north latitude, 125 degrees east longitude, which is public owned land in Okinawa Japan [20]. No protected species were involved in sample collections which were not required for permission. The crude CnP preparation II used in the *in vivo* study was extracted and purified as described previously [20]. Both the MCD diet, which was used as a pathological NASH model, and a methionine-choline-sufficient (MCS) diet, used as a control diet, were obtained in powdered form (Oriental Yeast Co., Ltd., Tokyo, Japan).

### Animal model and experimental design

Ten-week-old male *db/db* mice were purchased from Japan SLC Inc. (Hamamatsu, Japan). After a 1-week acclimatization period on a basal diet (Oriental Yeast), 17 mice were divided into three groups and fed one of the following diets for 8 weeks: (1) MCS, (2) MCD, or (3) MCD diet with CnP (1 μg/kg/d p.o.). The doses of CnP were determined in accordance with those used in a previous study [12]. All mice were given free access to water and experimental diets, and the body weight and food consumption of the mice in each group were recorded weekly. Protocols describing the use of mice were approved by the Institutional Animal Care and Use Committee of Aichi Medical University and were in accordance with the National Institutes of Health “Guide for the Care and Use of Laboratory Animals.” After being fed on the experimental diets for 8 weeks, the mice were euthanized by CO_2_ inhalation. Their livers were rapidly excised and then either fixed in buffered formalin (10%) or frozen in liquid nitrogen and stored at -80°C. Blood samples were collected from the left ventricle and centrifuged, and the serum was stored at -80°C.

### Serum and tissue biochemical measurements

Serum alanine aminotransferase (ALT) and fasting blood glucose (FBG) levels were determined using commercially available kits (Wako, Osaka, Japan), and serum immunoreactive insulin (IRI) levels were measured using a mouse insulin ELISA kit (Funakoshi, Tokyo, Japan). Stored liver samples (100 mg) were lysed and homogenized in 2 mL of a solution containing 150 mM NaCl, 0.1% Triton X-100, and 10 nM Tris using a polytron homogenizer (NS-310E; MicroTech Nichion, Tokyo, Japan) for 1 min. Hepatic TG and FFA contents were measured using a triglyceride detection kit (Wako) and free fatty acid detection kit (Wako), respectively.

### Measurement of malondialdehyde

Thiobarbituric acid reactive substances were measured in liver homogenates as a marker of oxidative stress. Liver samples were homogenized in cold 1.15% KCl buffer, and thiobarbituric acid reactive substances were determined using malondialdehyde (MDA) as a standard as previously described [21].

### Histopathological and immunohistochemical examination

Five-micrometer-thick sections of liver tissues originally fixed in formalin and embedded in paraffin were examined in all experiments. Hematoxylin-eosin and Sirius Red staining were performed to assess hepatic inflammation and fibrosis, respectively. Oil Red O staining was performed using a standard technique to assess hepatic fat deposition. Hepatic fibrosis was assessed from the percentage of the Sirius Red-positive area quantified by Image J, whereas the percentage of Oil Red O-positive area was measured using a computerized image analysis system with Image-Pro Plus version 4.5 (Media Cybernetics, Silver Spring, MD, USA). We also examined the steatosis-activity-fibrosis (SAF) score based on a histological scoring system for NAFLD described by Bedossa et al. [22]. The amount of steatosis (percentage of hepatocytes containing fat droplets) was scored as 0 (<5%), 1 (5%–33%), 2 (34%–66%), or 3 (>67%). Hepatocyte ballooning was classified as 0 (none), 1 (few), or 2 (many cells/prominent ballooning). Foci of lobular inflammation were scored as 0 (no foci), 1 (<2 foci per ×200 field), or 2 (2–4 foci per ×200 field). Hepatic activity grade is defined as the total of the ballooning and inflammation scores. Fibrosis was scored as stage F0 (no fibrosis), stage F1a (mild, zone 3, perisinusoidal fibrosis), stage F1b (moderate, zone 3, perisinusoidal fibrosis), stage F1c (portal/periportal fibrosis), stage F2 (perisinusoidal and portal/periportal fibrosis), stage F3 (bridging fibrosis), or stage F4 (cirrhosis) [22]. F1a, F1b, and F1c fibrosis were all scored as 1. These histological features were scored based on 10 randomly selected fields per section.

### Real-time polymerase chain reaction of liver RNA

Frozen liver specimens were crushed in TRIzol reagent (Life Technologies, Tokyo, Japan) and RNA extraction was performed using an RNeasy Mini Kit (Qiagen, Tokyo, Japan). The isolated RNA was resuspended in 40 μL of RNase-free water and quantified by spectrophotometry (optical density [OD] 260 and low-mass gel electrophoresis [Invitrogen, Tokyo, Japan]). The extracted total RNA was reverse transcribed to cDNA using a High Capacity cDNA Reverse Transcriptional Kit (Applied Biosystems, Foster City, CA) according to the manufacturer's instructions. Real-time quantitative PCR analysis of the RNA was carried out with the ABI Step One Sequence Detection System (Applied Biosystems) using TaqMan Gene Expression Assays (acyl-coenzyme A oxidase 1 [ACOX1], Mm01246834_m1; cluster of differentiation 36 [CD36], Mm00432403_m1; BCL2 associated X [Bax], Mm00432051_m1; carnitine palmitoyltransferase 1 [CPT1], Mm00463960_m1; microsomal triglyceride transfer protein [MTTP], Mm00435015_m1; sterol regulatory element binding transcription factor 1 [SREBF1], Mm00550338_m1; elongation of long chain fatty acids family member 6 [ELOVL6], Mm0085123_s1; fatty acid synthase [FASN], Mm00662319_m1; fatty acid transporter member 2 [FATP2], Mm0128768_m1; fatty acid binding protein 1 [FABP1], Mm00444340_m1; tumor necrosis factor-α [TNF-α], Mm00443258_m1; monocyte chemotactic protein-1 [MCP-1], Mm00441242_m1; PPARα, Mm00440936_m1; peroxisome proliferator-activated receptor γ [PPARγ], Mm01184322_m1; TGF-β, Mm00441724_m1; and tissue inhibitor of metalloproteinase 1 [TIMP1], Mm00441818_m1) and TaqMan Universal PCR Master Mix (Applied Biosystems) according to the manufacturer’s instructions. The detailed protocol for TaqMan PCR was determined based on a previous study [23].

### Statistical analysis

All results were expressed as means ± standard error (SE). Statistical analyses were performed using an analysis of variance (ANOVA). A P value of less than 0.05 was considered statistically significant, and if the overall P value was less than 0.05, Fisher's protected least significant difference post hoc test was performed to analyze differences between the multiple groups.

## Results

### Changes in body weight, hepatic steatosis, and hepatic TG and FFA contents

**Fig 1:**
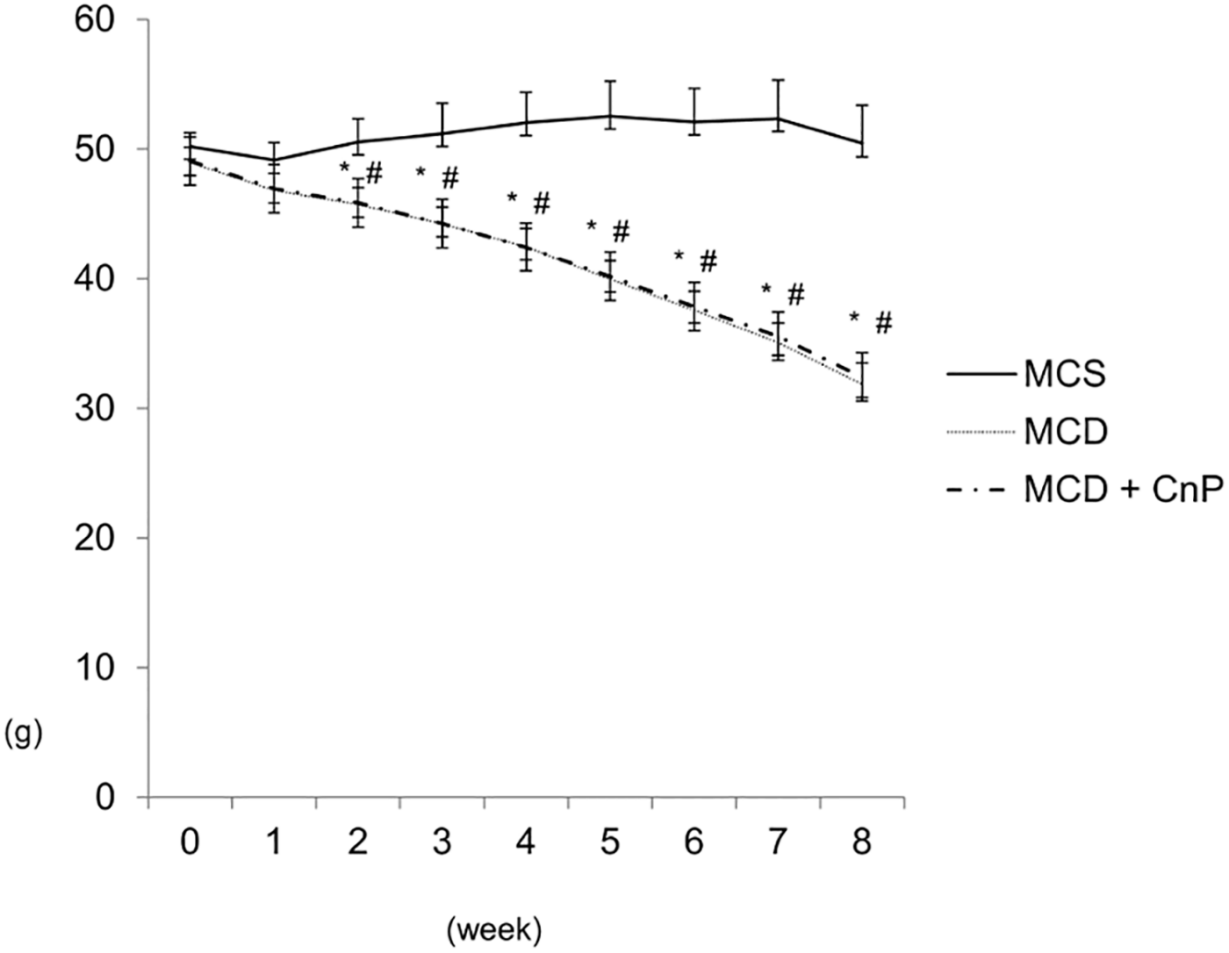
Time course changes in body weight. Mice were fed with a methionine-choline-sufficient (MCS, n = 5), methionine-choline-deficient (MCD, n = 6), or MCD with conophylline (CnP) (n = 6) diet. Statistical analysis was performed using ANOVA, and data are expressed as means ± SE. The overall P value was less than 0.05 from 2 weeks to 8 weeks (*P < 0.05, MCD diet compared to MCS diet; ^#^P < 0.05, MCD diet with CnP compared to MCS diet).

**Fig 2:**
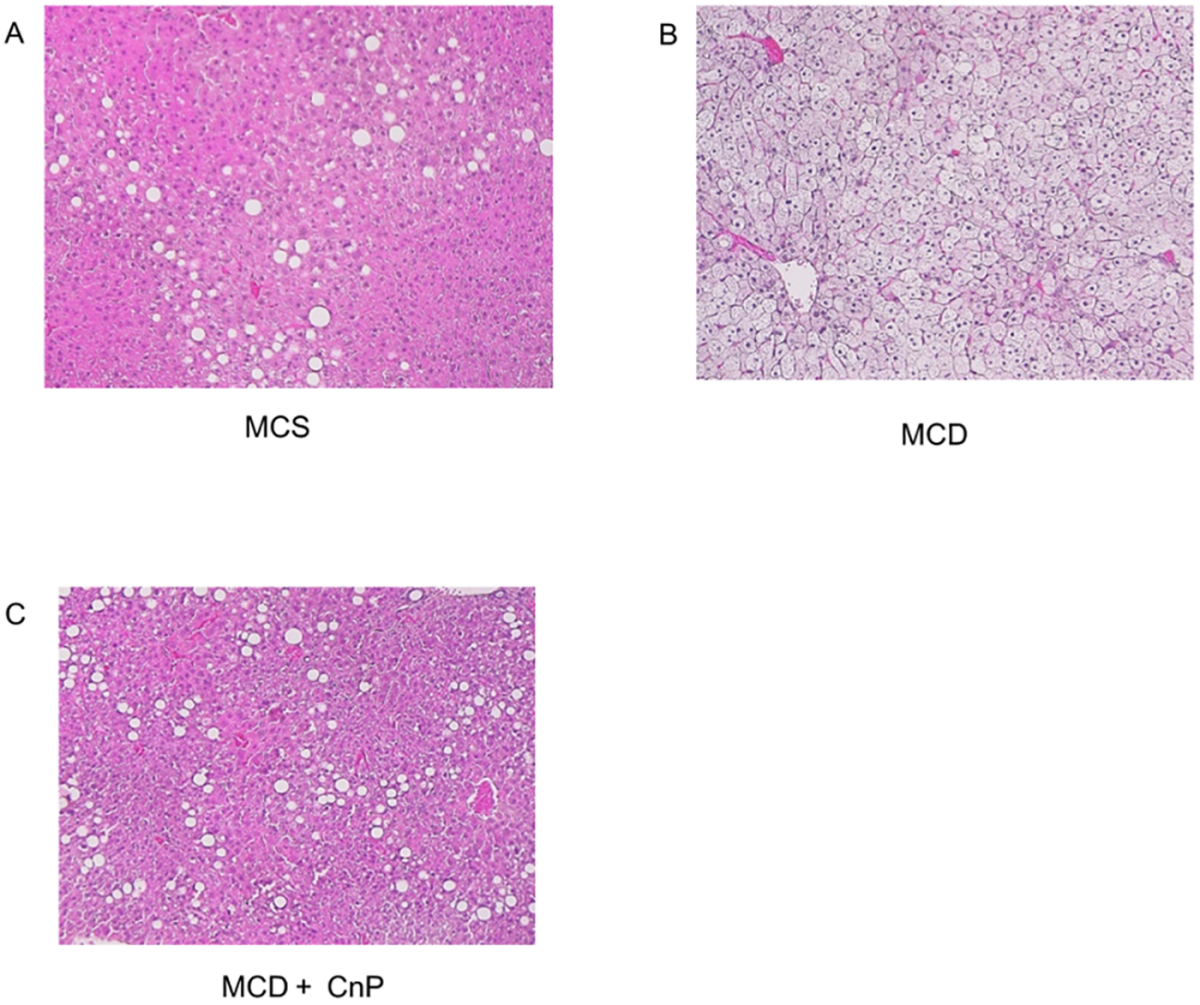
Representative images of the liver stained with hematoxylin-eosin. Mice were fed with a methionine-choline-sufficient (MCS) (A), methionine-choline-deficient (MCD) (B), or MCD with conophylline (CnP) (C) diet. Original magnification, ×100.

**Fig 3:**
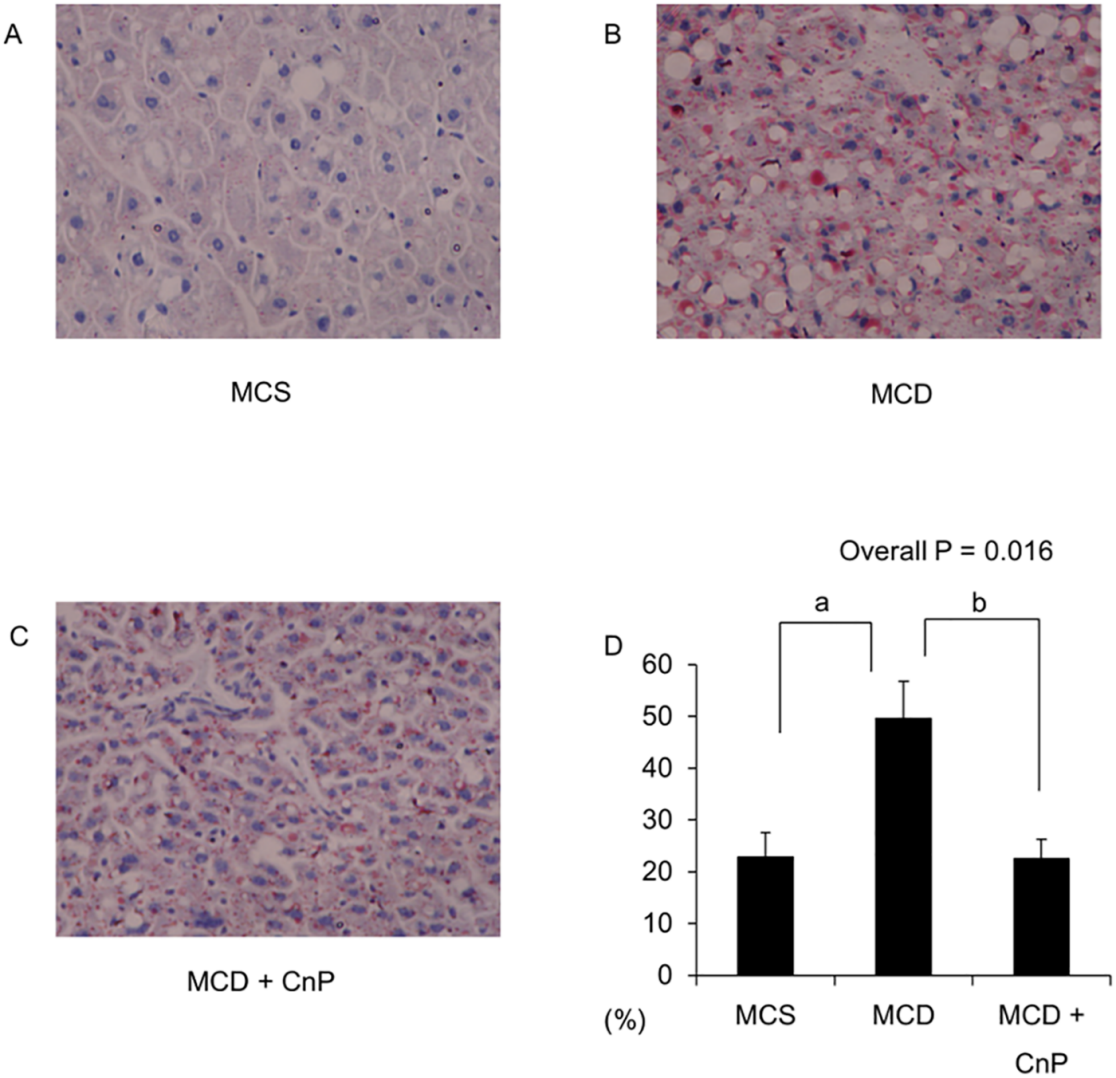
Representative images of liver stained with Oil Red O. Mice were fed with a methionine-choline-sufficient MCS (A), methionine-choline-deficient (MCD) (B), or MCD with conophylline (CnP) diet (C). (D) Quantitative analysis of changes in Oil Red O-positive area in the respective groups. Statistical analysis was performed using ANOVA, and data are expressed as means ± SE (^a^ P < 0.05 MCD diet compared to MCS diet, ^b^ P < 0.05 MCD diet with CnP compared to MCD diet). Original magnification, ×200.

Mice fed an MCS diet gained body weight, whereas those fed an MCD diet showed a reduced body weight gain that was not changed by CnP over the experimental period (Fig 1). The daily intake of the MCS diet was greater than that of the MCD diet, and this difference was not altered by the co-administration of CnP (Table 1). Hematoxylin-eosin staining showed that the MCD diet obviously increased hepatic steatosis, which was attenuated by CnP (Fig 2A–2C). Oil Red O staining also showed that the MCD diet obviously increased the Oil Red O-positive area, which was significantly inhibited by CnP (Fig 3A–3D). The MCD diet also significantly increased hepatic TG contents, steatosis scores, and hepatic FFA contents, which were significantly decreased by CnP (Tables 1 and 2). Compared with the MCD diet-fed mice, those mice fed the MCS diet showed significantly attenuated FBG and serum IRI levels, and these differences were not altered by co-administration of CnP with the MCD diet (Table 1)

**Table 1:** Clinical characteristics of mice fed experimental diets.

Group	n	Daily food consumption (g)	Hepatic TG (mg/g liver)	Hepatic FFA (mEq/L)	FBG(mg/dL)	IRI (μg/L)
**MCS**	5	6.0 ± 0.8	344 ± 77	1.2 ± 0.1	491 ± 49	3.4 ± 1.0
**MCD**	6	3.2 ± 0.5^a^	481 ± 84^a^	1.6 ± 0.2^a^	65 ± 11^a^	0.13 ± 0.04^a^
**MCD + CnP**	6	3.1 ± 0.8^a^	275 ± 46^b^	1.1 ± 0.1^b^	67 ± 11^a^	0.44 ± 0.16^a^

MCS, methionine-choline-sufficient diet; MCD, methionine-choline-deficient diet; CnP, conophylline; TG, triglyceride; FFA free fatty acid; FBG, fasting blood glucose; IRI, serum immunoreactive insulin

Data were analyzed using ANOVA. The overall P values for daily food consumptions, hepatic TG contents, hepatic FFA contents, FBG, and IRI levels were less than 0.05. Values represent means ± SE.

^a^ P < 0.05 MCD diet compared to MCS diet

^b^ P < 0.05 MCD diet with CnP compared to MCD diet

**Table 2:** Steatosis-activity-fibrosis (SAF) scores of mice fed experimental diets.

Group	n	Steatosis	Activity grade	Fibrosis
**MCS**	5	1.06 ± 0.29	0.38 ± 0.23	0.20 ± 0.08
**MCD**	6	2.10 ± 0.32^a^	2.3 ± 0.40^a^	1.55 ± 0.12^a^
**MCD + CnP**	6	1.23 ± 0.18^b^	1.02 ± 0.34^b^	0.62 ± 0.16^b^

MCS, methionine-choline-sufficient diet; MCD, methionine-choline-deficient diet; CnP, conophylline

Data were analyzed using ANOVA. Values represent means ± SE. The overall P values for steatosis, activity grade, and fibrosis were less than 0.05.

^a^ P < 0.05 MCD diet compared to MCS diet

^b^ P < 0.05 MCD diet with CnP compared to MCD diet

### Changes in hepatic lipid metabolism-related gene expressions

**Fig 4:**
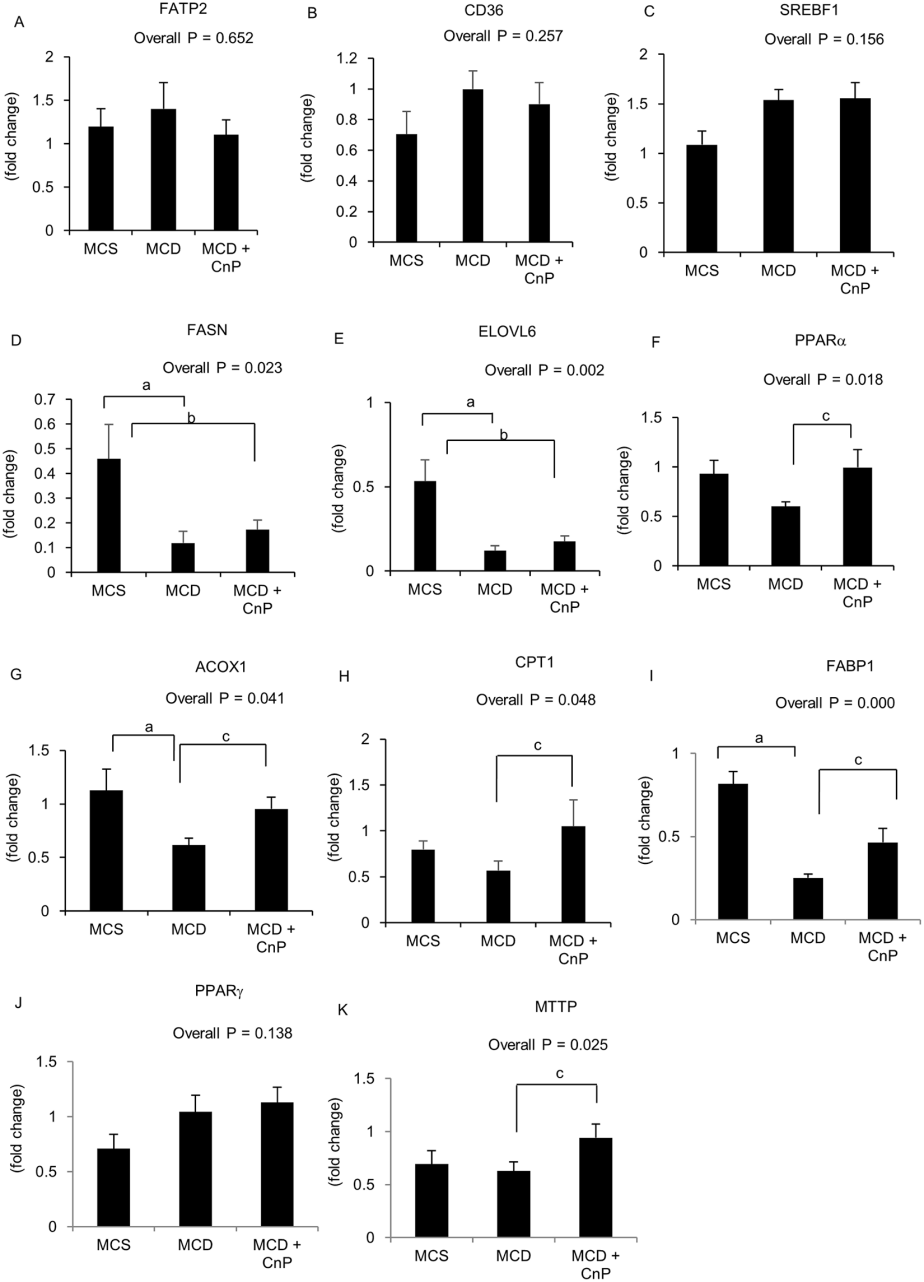
Evaluation of hepatic lipid metabolism-related genes. Mice were fed with a methionine-choline-sufficient (MCS), methionine-choline-deficient (MCD), or MCD with conophylline (CnP) diet. The relative mRNA expressions of FATP2 (A), CD36 (B), SREBF1 (C), FASN (D), ELOVL6 (E), PPARα (F), ACOX1 (G), CPT1 (H), FABP1 (I), MTTP (J), and PPARγ (K) were evaluated. Statistical analysis was performed using ANOVA, and data are expressed as means ± SE (^a^ P < 0.05 MCD diet compared to MCS diet, ^b^ P < 0.05 MCD diet with CnP compared to MCS diet, ^c^ P < 0.05 MCD diet with CnP compared to MCD diet).

The MCD diet did not change hepatic FATP2, but tended to increase hepatic CD36 mRNA levels, which were not changed by CnP (Fig 4A and 4B). Although hepatic SREBF1 mRNA levels were not changed by the MCD diet, hepatic FASN and ELOVL6 mRNA levels were significantly attenuated by this diet, and were not changed by CnP (Fig 4C–4E). The MCD diet tended to decrease hepatic PPARα mRNA levels, which were significantly increased by CnP (Fig 4F). Similarly, the MCD diet significantly decreased hepatic ACOX1 mRNA levels, which were significantly reversed by CnP (Fig 4G). Hepatic CPT1 mRNA levels likewise tended to be decreased by the MCD diet, and were significantly augmented by CnP (Fig 4H). The same MCD diet significantly attenuated FABP1 mRNA levels, which again were significantly increased by CnP (Fig 4I). In contrast, the MCD diet had no effect on hepatic PPARα mRNA levels, which were not changed by CnP (Fig 4J), whereas although the MCD diet did not change hepatic MTTP mRNA levels, these were significantly increased by CnP (Fig 4K).

### Changes in serum ALT levels, hepatic inflammation, and fibrosis, and their related gene expressions

**Fig 5:**
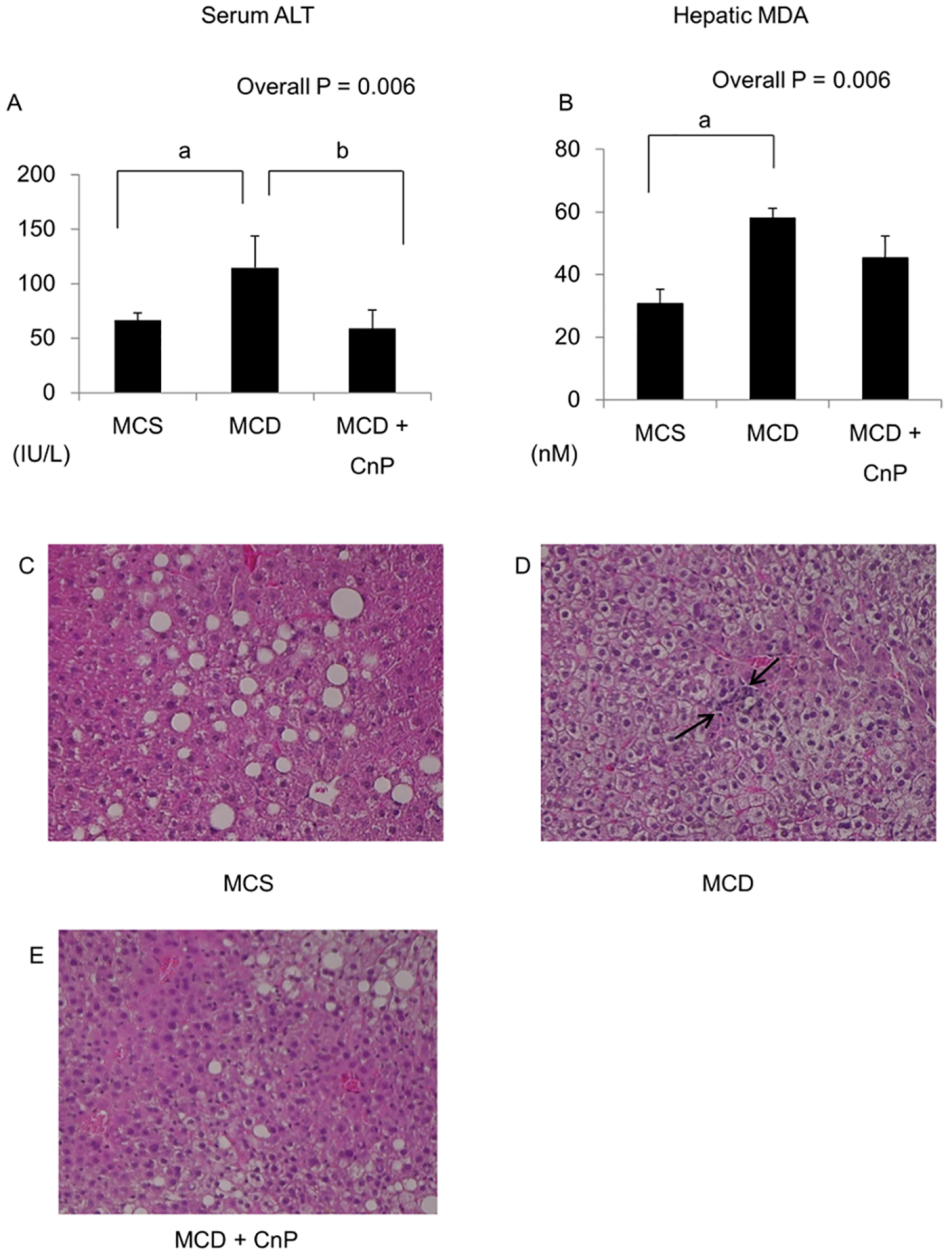
Evaluation of hepatic inflammation-related parameters. Mice were fed with a methionine-choline-sufficient (MCS), methionine-choline-deficient (MCD), or MCD with conophylline (CnP) diet. (A) Serum alanine aminotransferase (ALT) levels. (B) Hepatic malondialdehyde (MDA) levels. Representative images showing the inflammatory foci for MCD-induced liver injury in mice (C: MCS, D: MCD, and E: MCD diet with CnP). Original magnification, ×200. The arrows indicate the inflammatory cells in the liver. (F) The number of inflammatory foci per ×20 field was enumerated for each section. Statistical analysis was performed using ANOVA, and data are expressed as means ± SE (^a^ P < 0.05 MCD diet compared to MCS diet, ^b^ P < 0.05 MCD diet with CnP compared to MCD diet).

**Fig 6:**
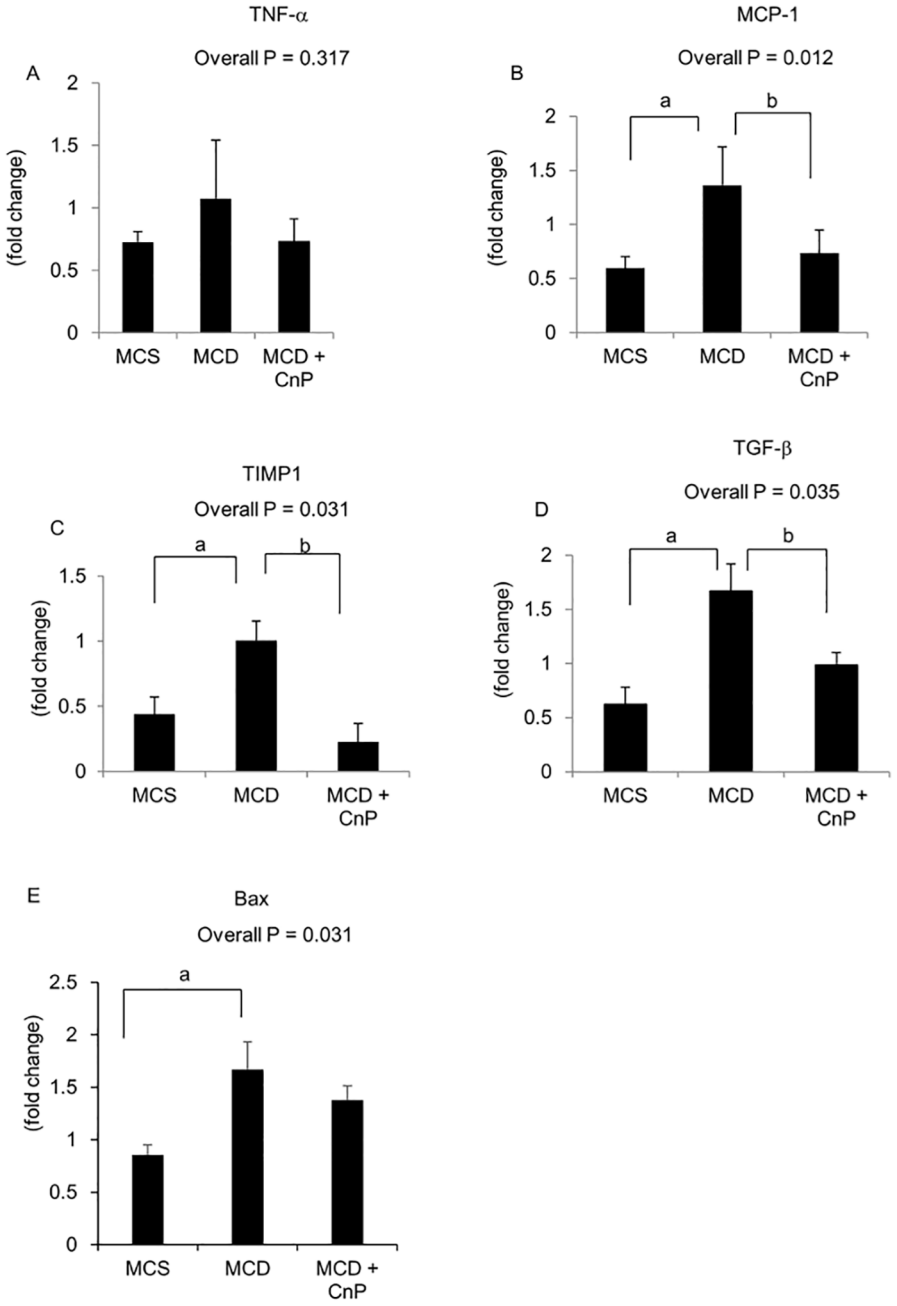
Evaluation of hepatic inflammation-related genes. Mice were fed with a methionine-choline-sufficient (MCS), methionine-choline-deficient (MCD), or MCD with conophylline (CnP) diet. Relative mRNA expressions of TNF-α (A), MCP-1 (B), TGF-β (C), TIMP1 (D), and Bax (E) were evaluated in the liver. Statistical analysis was performed using ANOVA, and data are expressed as means ± SE (^a^ P < 0.05 MCD diet compared to MCS diet, ^b^ P < 0.05 MCD diet with CnP compared to MCD diet).

**Fig 7:**
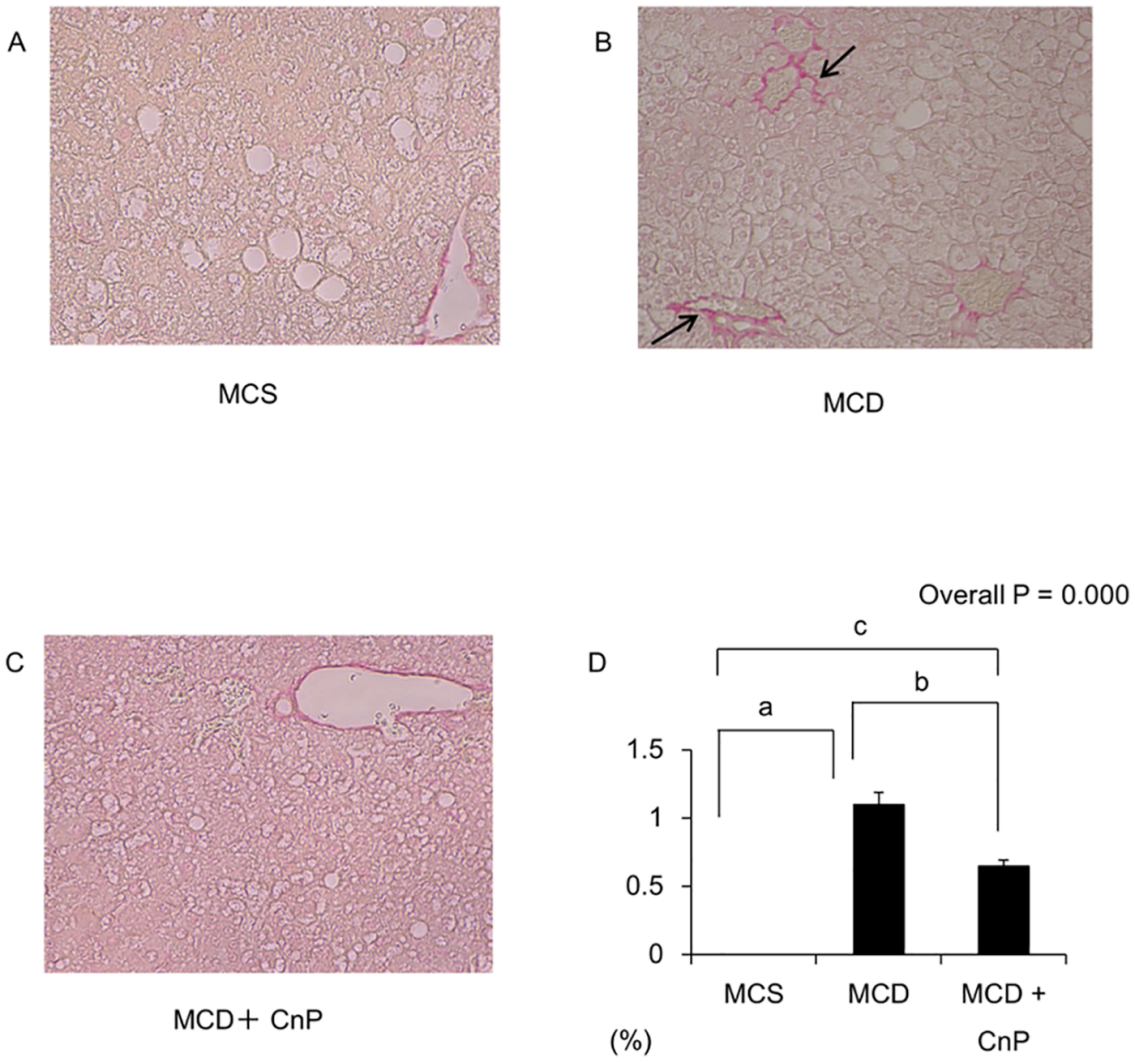
Representative images of the liver stained with Sirius Red. Mice were fed with a methionine-choline-sufficient (MCS) (A), methionine-choline-deficient (MCD) (B), or MCD with conophylline (CnP) (C) diet. (D) Quantitative analysis of changes in Sirius red-positive area in the respective groups. The arrows indicate the fibrotic collagen fibers in the liver, Original magnification, ×200. Statistical analysis was performed using ANOVA, and data are expressed as means ± SE (^a^ P < 0.05 MCD diet compared to MCS diet, ^b^ P < 0.05 MCD diet with CnP compared to MCD diet, ^c^ P < 0.05 MCD diet with CnP compared to MCS diet).

The MCD diet significantly increased serum ALT levels, which were significantly attenuated by CnP, and hepatic MDA levels, which tended to be decreased by CnP (Fig 5A and 5B). In contrast to the MCS diet, which did not induce hepatic inflammation, the MCD diet induced hepatic inflammation, which was attenuated by CnP (Fig 5C–5E). The MCD diet significantly increased hepatic activity grades, which were decreased by CnP (Table 2), and also tended to increase hepatic TNF-α mRNA levels, which tended to be decreased by CnP (Fig 6A). The MCD diet significantly increased hepatic MCP-1, TIMP1, and TGF-β mRNA levels, which were all significantly attenuated by CnP (Fig 6B–6D). In contrast, although the MCD diet significantly increased hepatic Bax mRNA levels, these were not changed by CnP (Fig 6E). Furthermore, whereas the MCS diet did not induce a Sirius Red-positive area (Fig 7A), the MCD diet did induce a Sirius Red-positive area, which was decreased by CnP (Fig 7B–7D). Finally, the MCD diet significantly augmented fibrosis scores, which were attenuated by CnP (Table 2).

## Discussion

In the current study, we investigated whether CnP inhibits MCD-induced hepatic steatosis, inflammation, and fibrosis in a NASH model. We demonstrated that administration of CnP attenuated MCD-induced hepatic steatosis as well as hepatic TG and FFA contents. Furthermore, the MCD-induced increases in hepatic inflammation and Sirius red-positive fibrotic area were also attenuated by CnP.

Although there are many animal models of NASH, a consensus regarding the optimal model is lacking. A recent report indicated that the metabolic profile associated with human NASH is mimicked by a Western diet (45% energy from fat, predominantly saturated fat, with 0.2% cholesterol, plus drinking water supplemented with fructose and glucose); however, NASH is less severe and less reproducible in the Western diet model [24]. In contrast, an MCD diet is a widely used and highly reproducible model of accelerated hepatic steatosis, inflammation, and fibrosis in the context of NASH, despite the associated loss of body weight and insulin resistance [18]. Moreover, the various mechanisms implicated in NASH pathogenesis, such as oxidative stress and lipotoxicity, are more severe in the MCD diet model than those in the Western diet model.

Emerging evidence has suggested that FFA is an important source of hepatic TG contents [25]. In the current study, hepatic FFA tended to increase in mice fed an MCD diet, and CnP significantly decreased MCD-induced FFA augmentation in the liver. The fatty acid transport protein CD36 is reported to be involved in FFA uptake in the liver [18, 26], and whereas FATP2 mRNA levels were not changed in MCD-fed mice, CD36 mRNA levels tended to increase. However, CnP had no affect hepatic FATP2 and CD36 mRNA levels, indicating that it does not affect FFA uptake in the liver.

With regards to hepatic *de novo* lipogenesis, the MCD diet did not change hepatic SREBF1, despite the downregulation of its downstream targets, such as FASN and ELOVL6. This apparent discrepancy could be explained either by a prominent post-transcriptional regulation of SREBF1 or by the involvement of upstream regulators different from SREBF1. FASN is reported to be downregulated independent of SREBF1 [27], indicating that an MCD diet downregulates hepatic *de novo* lipogenesis, which is not affected by CnP. Fatty acid binding protein (FABP) is involved in fatty acid intracellular transport in the liver, whereas ACOX1 and CPT1 are involved in β-oxidation in the liver [28–30]. The MCD diet administered in the present study significantly decreased hepatic FABP1 mRNA levels, which were recovered by CnP. Similarly, this diet significantly attenuated ACOX1 mRNA levels and tended to decrease CPT1 mRNA levels, which were also recovered by CnP. PPARα is expressed in the liver and involved in hepatic lipid metabolism [31].

The administration of PPARα agonists have been shown to attenuate MCD-induced hepatic TG accumulation [32], and to upregulate the mRNA of liver FABP and β-oxidation enzymes, thereby reducing hepatic TG and preventing steatosis in mice [33]. In the present study, we showed that an MCD diet tended to decrease hepatic PPARα mRNA levels, which were significantly increased by CnP. Impairment of the synthesis and release of very low density lipoprotein (VLDL), which plays an important role in hepatic TG excretion, might be a key factor for the progression of human NASH [34]. In this regard, MTTP, which is an important regulator of TG excretion in hepatocytes, is known to be involved in the production of VLDL [35]. In the present study, hepatic MTTP mRNA levels were not obviously changed by an MCD diet, and CnP significantly augmented MTTP mRNA levels in this model. Stimulation of hepatic intracellular fatty acid transport, β-oxidation, and excretion of fatty acid might be involved in reducing steatosis in the liver.

We found that an MCD diet significantly augmented hepatic inflammation and fibrosis concurrently with an increase in hepatic TNF-α, TGF-β, MCP-1, and TIMP-1 mRNA levels and hepatic MDA levels. The altered abundance and composition of fat in the liver might modulate the biological activity of Kupffer cells together with augmenting hepatic inflammation in MCD-fed mice [36]. Increased Kupffer cell activity has been shown to augment oxidative stress, which in turn stimulates the secretion of pro-inflammatory cytokines and chemokines such as TNF-α and MCP-1 [37], and TNF-α has been reported to directly augment TIMP-1 expression in hepatic stellate cells in vitro [38]. It has also been demonstrated that CnP inhibits TGF-β-induced apoptosis in rat hepatoma cells [39], and, consistently, we showed that CnP significantly decreased hepatic TGF-β mRNA levels, along with those of MCP-1 and TIMP-1. Although CnP also tended to reduce hepatic MDA levels, the Bax hepatic apoptosis stimulating genes were not changed by CnP. Previous reports have indicated that reduced TGF-β signaling attenuates hepatic apoptotic cells, but that CnP stimulates hepatic stellate cell apoptosis. [12, 40]. Thus, there is still a lack of agreement on whether CnP attenuates hepatic apoptosis, resulting in a decrease in serum liver enzymes and inflammation.

On the basis of the interrelationship between TGF-β and lipid metabolism, TGF-β signaling is reported to be associated with gene expression involved in lipogenesis and β-oxidation in fatty liver disease [40]. It has been shown that a choline-deficient diet significantly increases hepatic ACOX1 and CPT1 gene levels, and reduces choline-deficient diet-induced lipotoxicity in TGF-β receptor-deficient mice [40]. These results lead us to speculate that CnP reduces hepatic TGF-β signaling, probably contributing to the stimulation of β-oxidation-related genes. This stimulation of β-oxidation may in turn reduce hepatic lipotoxicity, resulting in decreased inflammation in the liver.

A previous study has demonstrated that CnP directly interacts with ARL6IP, which is an endoplasmic reticulum (ER) integral membrane protein [41]. ARL6IP is predominantly localized in the intracytoplasmic membranes and has been suggested to be involved in protein transport, membrane trafficking, or cell signaling during differentiation. However, the functional role of the ARL6IP protein and the responsive site of CnP in the liver remain to be elucidated.

In conclusion, the data obtained in the present study suggest that CnP attenuates steatohepatitis, possibly through the inhibition of TGF-β signaling and the stimulation of PPARα, FABP, and ACOX1-related β-oxidation in the liver. Reduced hepatic FFA might decrease lipotoxicity, resulting in the attenuation of inflammation and fibrosis. CnP could accordingly be considered as a therapeutic option to prevent hepatic steatohepatitis in NASH.
